# Insights into Thymus Development and Viral Thymic Infections

**DOI:** 10.3390/v11090836

**Published:** 2019-09-09

**Authors:** Francesco Albano, Eleonora Vecchio, Maurizio Renna, Enrico Iaccino, Selena Mimmi, Carmen Caiazza, Alessandro Arcucci, Angelica Avagliano, Valentina Pagliara, Giuseppe Donato, Camillo Palmieri, Massimo Mallardo, Ileana Quinto, Giuseppe Fiume

**Affiliations:** 1 Department of Experimental and Clinical Medicine, University “Magna Graecia” of Catanzaro, 88100 Catanzaro, Italy (E.V.) (E.I.) (S.M.) (C.P.) (I.Q.); 2 Institute for Endocrinology and Oncology, National Research Council, 80131 Napoli, Italy; 3 Department of Molecular Medicine and Medical Biotechnologies, University of Napoli “Federico II”, 80131 Napoli, Italy (M.R.) (C.C.) (M.M.); 4 Department of Public Health, University of Napoli “Federico II”, 80131 Naples, Italy (A.A.) (A.A.); 5 Department of Medicine, Surgery and Dentistry, University of Salerno, 84081 Salerno, Italy; 6 Department of Health Sciences, University “Magna Graecia” of Catanzaro, 88100 Catanzaro, Italy

**Keywords:** thymus, viral infections, T-cell development

## Abstract

T-cell development in the thymus is a complex and highly regulated process, involving a wide variety of cells and molecules which orchestrate thymocyte maturation into either CD4^+^ or CD8^+^ single-positive (SP) T cells. Here, we briefly review the process regulating T-cell differentiation, which includes the latest advances in this field. In particular, we highlight how, starting from a pool of hematopoietic stem cells in the bone marrow, the sequential action of transcriptional factors and cytokines dictates the proliferation, restriction of lineage potential, T-cell antigen receptors (TCR) gene rearrangements, and selection events on the T-cell progenitors, ultimately leading to the generation of mature T cells. Moreover, this review discusses paradigmatic examples of viral infections affecting the thymus that, by inducing functional changes within this lymphoid gland, consequently influence the behavior of peripheral mature T-lymphocytes.

## 1. Introduction

The thymus gland is a primary lymphoid organ, in which the essential development of T cells occurs. Within the thymus, hematopoietic precursors, deriving from bone marrow, enter the route of differentiation that will generate αβ- or γδ- mature T cells. Finally, these cells will exit the thymus and will colonize secondary lymphoid organs, where they will provide immunity against specific antigens.

The murine thymus has two lobes that are divided into multiple lobules by connective tissue, made of fibroblasts, collagen, and elastic fiber, as does the human thymus. The basic structural unit of the thymus is the lobule, composed of two distinct areas, the cortex and the medulla [1]. Each lobe has an outer cortex and an inner medulla. The cortex is densely populated by immature T-lymphocytes, while the medulla contains only a few mature T-lymphocytes.

**Figure 1:**
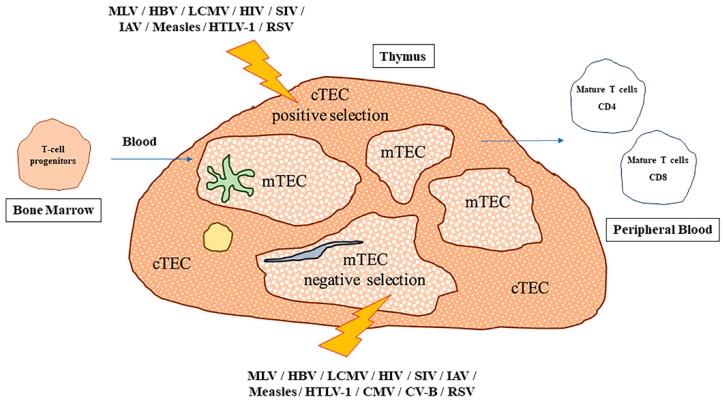
Impact of viral infections on intra-thymic T cell development. Two major populations of thymic epithelial cells (TECs) are present in the thymic parenchyma. In particular, cortical TEC (cTEC) cells are identified in the cortical compartment of the parenchyma, whereas medullary TEC (mTEC) cells are located internally. Dendritic cells (DCs, shown in green), fibroblasts (blue) and macrophages (yellow), contribute to the tissue organization. In humans, during the maturation process, bone marrow-derived T-cell progenitors arrive in the thymus via the bloodstream, where cTEC cells contribute to positive selection maturation, and mTEC cells contribute to the negative selection process, respectively. Also, DCs are likely responsible for negative selection in the cortex. Once the selection has been successfully completed and self-reactive cells have been removed by apoptosis, mature CD4^+^ or CD8^+^ T cells leave the thymus to populate the peripheral blood and secondary lymphoid organs. Viral infections are able to affect thymus functionality in multiple ways, but the most severe effects are observed when positive or negative selection is impaired, as these processes drive key steps of T-cell development. In the figure, lightning bolts indicate which viruses can specifically target and influence the activity of cTEC or mTEC populations.

The thymus medulla contains some Hassall’s corpuscles that are populated by eosinophilic type-VI epithelial reticular cells arranged concentrically, containing keratohyalin and bundles of cytoplasmic fibers (Figure 1). These cells differentiate from medullary thymic epithelial cells, following the inactivation of the autoimmune regulatory (*AIRE*) gene.

A third compartment can be found in the thymus, defined as the perivascular space (PVS). This compartment, despite surrounding blood vessels within the capsule, is physically separated from the thymic epithelial space. The role of the PVS has been often overlooked, due to its minimal extension during infancy and because it does not appear to be a functional site actively involved in thymopoiesis. However, as the thymus ages, the PVS enlarges and progressively replaces the epithelial area [2].

Thymic natural killer (NK) cells arise from early thymocyte precursors, and their development strictly relies on Interleukin-7 (IL7) signaling and requires the transcription factor GATA-3 [3,4,5]. Thymic NK cells are cytotoxic and characterized by the expression of CD127 (IL-7 receptor α). CD1-d restricted invariant Natural Killer T (iNKT) cells are a major producer of cytokines within the thymus, such as IFNγ and IL-4. Specifically, IL4, produced in combination with IL-13, triggering type-2 IL-4R signaling, is a key regulator of the migration during development [6].

In mice, three subsets of iNKT cells (NKT1-2-3) have been characterized. NKT2 are responsible for the IL-4 secretion, reside in the thymic medulla, and secrete cytokines even after infection in order to regulate and induce the development of the surrounding immune cells [7]. Thymic NK cells are, indeed, essential for the modulation of thymopoiesis and the maintenance of thymic architecture, and, also have a role in dendritic cell (DC) maturation [8].

Interestingly, the thymus contains macrophages and dendritic cells that originate from the bone marrow and arrive in the gland through blood vessels. More specifically, at least three different populations of macrophages have been identified within the thymus: dendritic, round, and flat-shaped cells (Table 1). Dendritic macrophages are randomly distributed throughout the thymus and are positive to markers Mac-2 and F4/80; flat-shaped macrophages (F4/80^+^, CD32/16^+^, Mac-2^−^) are distributed under the thymic cortex capsule; small oval macrophages, identified using the anti-Mac-2 antibody, are distributed in the thymic medulla and corticomedullary region (CMR) [9,10]. Thymic dendritic cells can be subdivided into conventional DC (cDC) (CD11c^hi^, MHC II^+^, CD45RA^−^) and plasmacytoid DC (pDC) (CD11c^medium^ MHC II^low^, CD45RA^high^, CD45R^high^). Moreover, conventional DCs can be further subdivided into CD8α^+^ and signal regulatory protein α+ (SIRPα+) cDC [11,12] (Table 1).

About 0.3% of thymic cells are B cells, a proportion that is comparable to the dendritic cells (Table 2). Thymic B cells are mainly located within the medulla and at the cortico–medullary junction. Some evidence suggests that they could develop intra-thymically, do not re-circulate and, harbor a distinct phenotype in comparison to peripheral B cells. Early disruption of T-cell development [23], as reported for TCRβ^−/−^ or CD3^−/−^ knock-out mice [23], as well as the disruption of the Notch signaling pathway, leads to an outgrowth of thymic B-cell progenitors and mature thymic B cells [24,25]. However, by using a murine model in which Cpa3-Cre causes deletion of Notch1 during the Double Negative 1–2 (DN1-DN2) stage of early T-cell development, Feyerabend et al. showed that thymic B cells deriving from the disruption of Notch signaling are phenotypically distinct from the B cells observed in the thymus of wild-type mice [26]. In addition, most of the cells that phenotypically resembled wild-type mature thymic B cells carried a functional copy of Notch. Hence, these data could indicate that upon a block of T-cell differentiation, T-cell progenitors do not simply switch toward a thymic-B cell fate, and that thymic T- and B-cell lineages diverge very early, even before the DN1-2 stage of early T-cell development [26,27]. Conversely, Yamano et al. more recently demonstrated that thymic B cells are simply thymus-migrated cells that participate in antigen presentation [28].

The thymic parenchyma provides the mechanical support, and includes immune and epithelial cells, such as lymphocytes, dendritic macrophages, dendritic cells, fibroblasts, matrix molecules (laminin, fibronectin, collagen, lectins), and hormones (thymulin, thymopoietin, thymosin-α1). Depending on the localization, in the thymic cortex or medulla, thymic epithelial cells (TECs) (CD45^−^, EpCAM^+^) are classified as cortical or medullary TECs (cTECs and mTECs, respectively). Specific progenitors for medullary TECs (mTECs) and cortical TECs (cTECs), as well as common medullary/cortical bipotent progenitors, have been identified from embryonic mouse thymus [13,14,15,16].

mTEC progenitors were initially identified by Rodewald et al., showing how the murine thymic medulla owns small groups of TECs (islets), each deriving from a single progenitor [13]; whereas, Bleul et al. and Rossi et al. showed the existence of medullary/cortical bipotent TEC progenitors even in the adult thymus [15,17].

More recently, rare sphere-forming cells (FoxN1^−^) have been identified in thymic stromal cells of the adult mouse thymus. These cells display several stemness features, such as cycling rate, self-renewal, and bi-potency, and could give rise to cTEC and mTEC lineages, or both (Table 1) [19]. 

cTECs (CD45^−^, EpCAM^+^, Ly51^+^, K8^+^, K5^−^, K14^−^) express a variety of self-antigens presented by the major histocompatibility complex (MHC), which determine the diversity of the T-cell repertoire through the process of positive selection. On the other hand, mTECs (CD45^−^, EpCAM^+^, Ly51^−/low^, CD80^high^, K8^−^, K5^+^, K14^+^) express a different set of self-antigens producing a self-tolerant T-cell repertoire through the elimination (so-called “negative selection”) of autoreactive T cells that bind to medullary self-antigens with high affinity, through the process defined as “central tolerance”. Moreover, mTECs are important constituents of Hassall’s corpuscles and regulate the production of regulatory Tvcells (T_reg_s) (CD4^+^, CD25^+^, Foxp3^+^), which dampen immune responses in peripheral tissues through the process of “peripheral tolerance” [18]. In this context, we can find thymic nurse cells (TNCs), that are specialized epithelial cells, expressing cytokeratins 5 and 8 (K5 and K8), which reside in the thymic cortex and are characterized by the expression of surface marker pH91. These cells are able to incorporate up to 200 lymphocytes within specialized cytoplasmic vacuoles, and it is thought that they play a pivotal role in T-cell development and MHC restriction [20,21,22]. Finally, as the thymus originally develops from an endocrine area of the foregut, its cells are still able to produce some hormones, such as thymulin, thymosin, thymopentin, and thymus humoral factor, which can regulate thymic physiology [39].

Besides providing an initial overview of the structure, cellular organization and composition of the thymus, in this review we describe in detail, and analytically discuss, the process of thymocyte development into mature T cells, which includes the positive and negative selection steps, highlighting the role of some key transcriptional factors, in both humans and mice. Finally, we provide some paradigmatic examples of viral infections impacting the thymus in the first instance, and ultimately leading to aberrant behavior of peripheral mature T cells in patients. The potential of therapeutic strategies aimed at boosting thymopoiesis, in the context of viral infection of human pathogens, is discussed.

## 2. Thymocyte Development and Related Transcription Factors

The process of the T-cell maturation in the thymus represents one of the major events leading to the functional and effective development of the entire immune system. Lymphocyte antigen receptors are generated by DNA recombination, ensuring these cells are able to recognize and bind a large panel of molecules of the most diverse provenience. In the lymphoid organs, T cells undergo sequential steps of maturation that ultimately lead to mature T cells [40]. Within the process, it is possible to discriminate between a first part, defined positive selection, which allows the survival of T cells expressing a competent receptor. Eventually, self-reactive T cells are discarded (during negative selection), generating a pool of mature and functional T cells.

In humans, immature T cells can express different T-cell antigen receptors (TCR) due to DNA rearrangements, however, in the thymic cortex, cells are positively selected as they are able to recognize host MHC molecules [41]. In the thymic cortex, the genes encoding for T-cell recombination-activating 1 and 2 proteins (*RAG1* and *RAG2*) are activated and operate the random recombination of V, D, and J segments, which in turn lead to T cells expressing both a pre-TCR (bearing α and β chains) and the co-receptors CD4 and CD8 (double-positive, DP stage). The TCRα chain rearrangement persists until a productive interaction between the TCRαβ complex and the MHC complex of the cTECs occurs and is recognized by the positive selection of the T cell. Thymocytes undergo apoptosis if they do not generate a productive TCR rearrangement; in fact, the latter can interact with peptides presented by the MHC, which are expressed by cells of the microenvironment. In this context, the interactions between the T cells and the cTECs determine the fate of thymocytes [16,17,19] (Table 1). During the positive selection stage, CD4^+^/CD8^+^ double positive (DP) thymocytes expressing TCRs poorly reactive against self-peptide:MHC complexes receive signals to survive, further differentiate, and give rise to CD4 or CD8 single positive (SP) cells, depending on their differential affinity toward either MHC class-II or class-I, respectively [32,33,34,35]. Such a process, which we refer to as “positive selection” requires both close contact and functional crosstalk with thymic stromal cells and occurs over a period of several days (Figure 1). During positive selection, the cTECs express a signature of self-antigens pivotal for the isolation of MHC-restricted T cells with functional TCR [42]. Among them, we can find Psmb11 (β5t), a subunit of the tymo-proteasome, a specialized protein complex whose activity is required for the generation of CD8-selection peptides [43]. On the other hand, cathepsin L and Prss16 (thymus-specific serine protease-Tssp) exert the same activity for the CD4 T cells [32]. Following these processes, the SP cells express the chemokine receptor CCR7, and they are attracted into the medulla where mTECs produce high amounts of the CCR7 ligands, CCL19 and CCL21 [44].

The other thymic population, mTECs, mediate the second essential step required for the maturation of T cells, negative selection. The cortical thymus homes T cells with productive rearrangements of the TCR chains. However, these cells are able to interact with both non-self-antigens or self-antigens, leading to potential auto-immunity events. Hence, these autoreactive cells have to be removed by apoptosis during the process of “negative selection” [45].

Stritesky et al. have reported that 75% of negatively selected cells are deleted, at the DP stage, in the thymus cortex, whereas the remaining 25% are deleted, at the SP stage, in the medulla [46]. These findings are in line with those previously reported by McCaughtry et al., according to which, the clonal deletion occurs in the thymus cortex by rare CD11c+ cortical dendritic cells, and elimination of such cells impairs negative selection [47].

As an alternative, they can be subjected to “receptor editing” in the thymus [48,49,50], even though Kreslavsky et al. denied such a mechanism [51]. The negative selection machinery thoroughly surveys the T-cell repertoire and supplies the first defense against the autoimmunity mechanisms. In addition, under circumstances whereby T cells escape from the central thymic control processes, they become anergic in the periphery, or apoptotic after activation [52,53], by means of the peripheral tolerance mechanism [48].

Upon positive selection, CD4 or CD8 T cells will express CCR7 and migrate into the medulla. Herein, these cells will interact with medullary thymic epithelial cells (mTECs), which express high levels of the CCL19 and CCL21 ligands; in the case of positive interaction, the deletion of autoreactive T cells will be promptly executed [26]. mTECs can be considered to be the learning-center for the T cells. Indeed, in order to mimic the expression of antigens that T cells could encounter in other tissues, they are able to express a great variety of tissue-restricted antigens (TRAs) [19]. By doing so, mTECs allow the deletion of cells that could recognize self-antigens that would otherwise be recognized in peripheral districts (Figure 1). 

Although the thymic medulla is essentially constituted by AIRE^+^ positive cells, it is characterized by substantial heterogeneity. Miller et al. and Bornstein et al. have recently provided further insights into such mTEC heterogeneity [54,55]. In particular, they identified a novel subset of mTECs, similar to peripheral tuft cells, which are usually associated with the intestinal mucosal barrier and express mediators of the taste transduction pathway and IL-25 [54,55]. The differentiation and the spatial localization of these thymic tuft cells were strongly dependent on (homeodomain interacting protein kinase 2) HIPK2, an AIRE-binding partner. In addition, they showed that thymic tuft cells promoted the development and polarization of iNKT2 within the thymus, establishing a type-2 thymic medullary microenvironment, enriched in IL4, Pou2f3 (a transcriptional factor, marker of a rare chemosensory cell type found in the gastrointestinal and respiratory tracts), and Trpm5 (mediator of the taste transduction pathway) [54].

Interestingly, medullary dendritic cells also contribute to the “tutoring” activity exerted by mTECs cells. As such, this represents a strategy to emphasize the importance of TRA production by mTECs [40]. Indeed, this mechanism allows the deletion, mediated by dendritic cells and mTECs, of almost all T cells recognizing self-antigen–MHC complexes [41]. Therefore, the process leading to the elimination (or clonal deletion) of autoreactive T cells is defined as “negative selection”. One can easily envisage how the process of T-cell selection is, in great part, regulated by TECs, which provide the microenvironment to test the cross-reactivity of the randomly generated T cells, well before they are committed to leaving the thymus. At this particular stage, these T cells will express a functional TCR without significant reactivity to self-antigens, and they are “authorized” from the thymus to migrate to secondary lymphoid organs (such as the spleen and lymph nodes) and eventually circulate throughout the body.

In mice, T-cell progenitors (Lin^−^, IL-7R^+^, Thy-1^−^, Sca-1^low^, c-Kit^low^) derive from hematopoietic stem cells of the bone marrow that migrate to the thymus via the bloodstream, and enter the thymus close to the cortico–medullary junction (Table 3). Here, they generate double negative DN1 thymocytes (CD117/c-KIT^+^, CD44^+^, CD25^−^) that evolve in DN2 (CD117/c-KIT^+^, CD44^+^, CD25^+^), and subsequently in DN3 (CD117/c-KIT^−^, CD44^−^, CD25^+^). DN3 cells migrate to the sub-capsular zone of the thymic lobules, where they undergo TCR rearrangements. The outward migration relies on the concerted actions of CCL25 and CXCL12/CXCR4 that contribute to thymocyte proliferation and differentiation towards DN4 (CD117/c-KIT^−^, CD44^−^, CD25^−^), and subsequently the double positive (DP) CD4^+^/CD8^+^ stage. In humans, three phenotypes of thymus-seeding progenitors (TSP) have been proposed: (a) CD34^high^, CD45RA^high^, and CD7^+^ cells [29]; (b) Lin^−^, CD34^+^, CD10^+^,and CD24^−^ cells [30]; and (c) Lin^−^, CD34^+^, CD10^−^, CD45RA^+^, and CD62L^high^ cells [31]. Haddad et al. reported that CD34^high^, CD45RA^high^, and CD7^+^ cells were able to migrate into thymic lobes in an ex vivo culture system [29]. The thymic progenitor differentiates into a DN compartment that is subdivided in DN1 (CD34^−^, CD38^−^, CD1a^+^), DN2 (CD34^−^, CD38^+^, CD1a^+^), DN3 (CD34^+^, CD38^+^, CD1a^+^), and DN4, also defined as immature single positive (CD3^−^, CD4^+^) [31].

During T-cell development, the TCR loci are rearranged, with a large majority of cells being converted into TCRαβ^+^ T cells. Moreover, different rearrangements at the Tcrδ, Tcrγ, and Tcrβ loci, starting at DN2 and completed at DN3, will lead to γδ-T cell lineage. This differs from TCRαβ^+^ T cells, which do require a peripheral activation in order to differentiate into effector cells; γδ-T cells are intrinsically programmed to differentiate into effector cells.

In mice, the β-selection specifically occurs at the DN3 stage, where the progenitor cells rearrange TCR loci to produce a functional β-chain; otherwise, development of the progenitor cells would not progress and they would die instead [36]. In humans, the exact point at which β-selection occurs is still controversial; it has been ascribed to the DP stage [37], the immature single positive stage [36,38], or the CD1a^+^ DN3 stage [56]. The pre-TCR and Notch signaling pathway act synergistically in DN3 thymocytes to trigger the β-selection process by, on one hand, reducing the gene expression of components of the RAG recombinase (*Rag1* and *Rag2*) and, on the other hand, upregulating transferrin receptor CD71 (Tfrc) and other trophic receptor genes [57]. As a consequence, thymocytes lose the cytokine receptor CD25 (IL-2Rα), becoming DN4 cells, which maturate into CD4^+^, CD8^+^ double positive (DP) cells through the acquisition of expression of TCR, CD4, and CD8. It is worth noting how, in its entirety, the DN cell compartment represents only 5% of total thymocytes, whereas the pool of DP cells constitutes 75–80% of thymocytes, thus representing the most abundant cellular component of the thymus.

Specific key transcriptional regulators control the development of T cells in the thymus, including Notch, Tcf-1, GATA-3, and Bcl11b (Table 4a). The initiation of the T-cell development program largely depends on Notch signaling, and the thymic cortical environment is indeed abundant in Notch ligand, mainly Delta-like ligand 4 (DLL4), which is strongly expressed by thymic stromal cells [58]. Upon engagement of Notch1 by Delta-like ligand 4, a proteolytic release of intracellular Notch1 occurs. Notch1 can translocate into the nucleus and binds and activates RBPJ, stimulating the expression of Notch target genes [59]. The first T-cell specific genes include *Tcf7* initially and, at a later stage, *GATA-3*, and also *Bcl11b*, which regulate, in coordination with the same Notch, T-cell specification and commitment fate [60,61,62].

During the initial phase of their development, T cells express PU.1, whose expression is completely abrogated by the repressor GATA-3 during lineage commitment [63]. In particular, GATA-3 is a pivotal transcription factor that drives T-cell development, acting as a potent repressor of B-cell potential, even at low expression levels [64]. GATA-3, by its ability to repress PU.1 and Pax5, can inhibit both the B-cell and myeloid developmental alternative paths [64]. Following the initial induction by Notch, GATA-3 expression is likely maintained by Myb and Tcf-1, both acting as positive regulators [65].

The progression of thymocytes toward the DN2 stage correlates with an increase of Bcl11b expression, depending on the action of Notch, with the likely additional contribution of other transcriptional factors (Table 4a) [66,67]. Bcl11b is required to complete the exclusion process, to restrict and limit the possibility of non-T-cell fates [66,67,68], and also to inhibit residual NK cell lineage potential in DN2 cells [69]. In particular, Bcl11b is able to decrease Kit expression, thus creating the DN2b phenotype, likely by means of a direct competitive binding to a potential Kit enhancer region [70].

The CD4^+^/CD8^+^ lineage dichotomy is also controlled by the ThPOK and Runx3 transcriptional factors, which exert opposite functions. ThPOK drives thymocyte development toward a CD4^+^ T_h_ fate and prevents the differentiation of thymocytes into CD8^+^ Cytotoxic T Lymphocytes (CTLs). Runx3 abolishes CD4 expression, inducing CTL-lineage differentiation instead. Other transcriptional factors play a pivotal role for the CD4^+^ lineage; they include two E-box binding proteins (E-proteins), namely E2A and HEB, GATA-3, and three transcriptional regulators belonging to the High Mobility Group family (Tox, Tcf1, and Lef) [71]. Proteins containing HMG-box domains are involved in a variety of physiologic protein–protein interactions and in various pathologic conditions, including cancer [72,73]. In addition, the action of ThPOK is critical even in already fully-differentiated, mature (peripheral) T cells, as it counteracts the cytotoxic fate of MHC class II-restricted CD4^+^ T cells, even when they differentiate into different T_h_ effector subsets [74]. Runx3 is essential for CD8^+^ lineage, and its expression is activated at the stage of CD8^+^ SP cells after positive selection and persists until the mature CD8^+^ T-cell stage. In addition, Runx1, Runx3, and the zinc-finger Mazr can inhibit the expression of ThPOK by binding specific silencer elements upstream of the ThPOK promoter (Table 4a) [75,76,77,78].

Recent findings report how NF-κB transcriptional activity is required for the later steps of T-cell maturation [79,80,81]. More specifically, a strong activation of NF-κB occurs at the semi-mature stage, after positive selection and before the migration of cells from the thymus. At this stage, the NF-κB activity is critical for protecting cells from complex-II mediated death downstream of TNF-R1 in the first instance. In addition, it sustains the maturation process so that thymocytes are made capable of mounting a proliferative response, when and if stimulated through the antigen receptor [79].

The organogenesis, development, and the age-related, progressive involution of the thymus are strictly dependent on TEC differentiation, homeostasis, and function. FoxN1 is the most important transcriptional factor for TEC development. FoxN1 belongs to the fork-head (FOX) superfamily of transcription factors (TFs), which are implicated in developmental defects in vertebrates. In the thymus, FoxN1 promotes the development of thymic epithelial progenitor cells (TEPC) into functional medullary thymic epithelial cells (mTECs) and cortical thymic epithelial cells (cTECs) [82,83], and maintains postnatal TEC homeostasis [84,85]. A lack of functional FoxN1 arrests the differentiation of TEPC into mTEC and cTEC [86]. Likewise, the transcription factor p63 has an essential function in the development of the thymus epithelium and epidermis [87], and is also required for the proliferation and differentiation of TEPC [87,88]. Two p63 isoforms are known: TAp63, containing the N-terminal transactivation domain, and ΔNp63, lacking this domain. ΔNp63 and FoxN1 are both highly expressed in the fetal thymus [89,90], while in the adult thymus TECs expressing FoxN1^+^ and ΔNp63^+^ TECs decrease with age [75,91,92].

The transcription factor AIRE is critical for thymus development, and is required for clonal deletion of self-reactive T cells [93]. The *AIRE* gene seems to be expressed mostly in a small population of mTEC (CD80^hi^ MHC-II^hi^) [94]. *AIRE* expression represents a hallmark for mTEC differentiation and can be regulated by several factors including methylation, RANKL-RANK-mediated NF-κB activation, the leukotriene β-mediated pathway, and perhaps miRNAs [94]. More specifically, *AIRE* is involved in the expression of specific tissue-restricted antigens (TRA) such as insulin, casein, and muscular acetylcholine receptor, as well as the expression of Xcl1, Ccr7, and Ccr4 ligands, which are essential for the differentiation and functionalization of mTEC. It has been reported that, albeit with a low affinity and no specificity toward any DNA sequence, *AIRE* binds to wide genome regions, including promoters characterized by the presence of epigenetic repressive markers (i.e., methylated H3K27) and the lack of permissive markers (methylated H3K4). On such promoters, *AIRE* contributes to the induction of the transcription elongation by binding to a variety of transcriptional factors and regulators, including Brd4 and Top1/2, and thus facilitating the recruitment of P-TEFb [93].

Recently, Takaba et al. have identified Fezf2 as a novel key transcriptional factor regulating the expression of TRAs in mTEC (Table 4b) [95]. Interestingly, Fezf2-dependent TRA genes are different from *AIRE*-dependent genes and include *Fabp9* (usually highly expressed in the testes), lipoprotein Apo-b and thrombin F2, well-known auto-antigens for which roles in different autoimmune disease, such as atherosclerosis and systemic lupus erythematosus, have been described [95]. Fezf2 is characterized by different DNA binding motifs, including one Eh1 domain and six C2H2-type zinc finger-domains [96]. However, the molecular mechanisms by which it regulates the transcription of TRA genes in mTEC remain to be elucidated.

## 3. Immunological Consequences of Viral Infections of the Thymus

The thymus is an organ commonly targeted by infectious pathogens such as viruses, bacteria, and fungi. Such infections may induce phenotypic and functional changes within the thymus, including alterations of proliferation, death, secretion, migration, and differentiation of thymocytes (Figure 1, Table 5). The behavior of mature, peripheral T-lymphocytes can be equally affected [97]. One of the most common effects on thymic function caused by pathogen infections is the impairment of the central tolerance process in thymocytes, through the impairment of both positive and negative selection processes. Nevertheless, the recruitment of antimicrobial immunity directly to the thymus can help to resolve local infection [98].

Tolerance to pathogens occurs frequently during viral infection. Specifically, it has been shown that infecting mice with thymotropic murine leukemia virus (MLV) very soon after birth can elicit T-cell tolerance to virus antigens [99,100]. Similarly, lymphocytic choriomeningitis virus (LCMV), following infection in mice, is primarily restricted to thymic CD4^+^ T cells, with only marginal involvement of cytotoxic CD8^+^ T cells, causing suppression of virus-specific T cell responses [101,102]. Such chronic infection could be eliminated by adoptive transfer of CD8^+^ T cells obtained from LCMV-immunized mice. Even more interestingly, the viral clearance induced the re-acquisition of LCMV-specific CTL responses, indicating that continuous presence of the antigens in the thymus can trigger tolerance [100,102,103]. Similar to MLV and LCMV infections, prenatal intrauterine infection with HBV (Hepatitis B virus) induces T-cell tolerance to viral proteins [126] and children born from HBV-infected mothers are more likely to become chronic carriers of HBV [127], suggesting once again how the presence of viral proteins within the thymus can induce an HBV-specific T-cell tolerance. Of note, both MLV and HBV are capable of infecting TEC cells [99,128], and the observation of a rapid turnover of TECs could likely support the hypothesis that these viruses can continually infect new TECs, or reside in thymic epithelial stem cells [100]. Altogether, these data indicate that direct thymic infections by viruses can severely alter T-cell selection and induce tolerance against invading pathogens, with the consequent effect of impairing ongoing immunity.

Takamura et al., have analyzed the effects of Friend virus (FV) infection on the thymus in mice [129]. Friend virus is a murine-tropic retrovirus constituted by two gamma-retroviruses, whose replication occurs mainly in fully dividing hematopoietic cells, which leads to the exhaustion of antigen-specific CD8^+^ T cells. They found that upon infection, FV spread to, and persisted within, the thymus, infecting and replicating mainly into DN thymocytes that will then proliferate and develop into DP cells. Interestingly, the viral antigens were expressed in all DC subsets as well as in mTECs and cTECs. In addition, they found that FV infection had no impact on the number and frequency of thymocyte populations, and also did not alter thymic architecture, yet caused the clonal deletion of FV-specific thymocytes through the direct deletion action of DC and cTEC cells expressing viral antigens. However, they showed that in FV-chronically-infected mice, FV-reactive CD8^+^ T cells, freshly primed upon chronic infection, were nevertheless able to differentiate into memory CD8^+^ T cells, with a stronger functionality, as compared to the exhausted memory CD8^+^ T cells at the peripheral level [129].

It is also worth underlining the evasion strategies of anti-viral CD8^+^ T-cell-mediated immunity adopted by murine retrovirus through the regulation of central and peripheral tolerance. In the context of FV infection, we can summarize the following points: (1) during the acute phase of infection, FV-infected-erythroblasts expressing PD-L1 induce the exhaustion of CD8^+^ T cells in the periphery; (2) FV spreads to the thymus and induces the expression of viral antigens on thymic DCs and TECs; (3) the presentation of viral antigens by DCs and TECs in the thymus blocks the production of pathogen-reactive naïve CD8^+^ T cells as source of functional memory CD8^+^ T cells.

Induction of virus-specific central tolerance is not a characteristic unique to the Friend virus. Besides the aforementioned mechanism, it has been reported that neonatal infection with either the Gross murine leukemia virus (G-MuLV) or the Moloney murine leukemia virus (MMLV) determines a strong viral replication within the thymus, ultimately inducing a life-long immune non-responsiveness to viral antigens [129].

Severe atrophy of the thymus is a common feature observed during several acute infections and involves mainly CD4^+^ CD8^+^ DP thymocyte subsets. The depletion of the double positive thymocyte (DP) compartment has been reported in a wide variety of bacterial, protozoal, and viral infections, including Human Immunodeficiency Virus-1, HIV-1 infection. Thymic structural and functional alterations have been previously reported in HIV infection [104]. Specifically, in HIV-1 infected infants, characterized by a fast onset of Acquired ImmunoDeficiency Syndrome, AIDS, evidence of thymic dysfunctions, related to an altered architecture of the thymus, have been found [105]. It is well known that HIV-1 and Simian Immunodeficiency Virus (SIV) are able to infect the thymus [106,107], thus inducing thymic alterations and clinically relevant conditions. Upon specific infections, atrophy of the thymus is highly frequent in patients and is usually characterized by a block in thymocyte maturation (from DN to DP thymocytes), ultimately leading to a progressive and massive reduction of DP and SP (CD4^+^, CD8^−^) thymocytes [105,108]. In HIV-1-infected infants, the strong decrease of CD4^+^ and CD8^+^ T cells in peripheral blood is caused by a massive reduction of thymic DP cells [109]; similar observations have been obtained in SIV-infected macaques [110,111]. Recently, our research group has developed and characterized a murine transgenic model (Tat-Tg mice), specifically expressing the HIV-1 Tat protein in the lymphoid tissues. Strikingly, this transgenic line was mainly characterized by a marked atrophy of the thymus, characterized by an increase of DN4 thymocytes subset coupled with a massive reduction of the DP subset. This phenotype showed typical features of pediatric and severe AIDS, suggesting that Tat represents the most important etiopathogenic viral protein involved in AIDS-related thymic atrophy [112]. More specifically, an aberrant increase of p65/NF-κB activity was observed in thymocytes derived from Tat-Tg mice, likely due to both the pro-activating interaction between Tat and p65 [130] and the inhibitory interaction with IκB-α [131,132,133], de-regulating the expression of several key cytokines/chemokines and microRNA-181a-1 and, thus, affecting thymocyte differentiation from DN4 toward DP. It is likely that the effects of these alterations resulted in an increased DN4 sub-population of the DN compartment, associated with the massive loss of peripheral CD4^+^ and CD8^+^ T cells. 

Interestingly, it has been shown how, following the infection of respiratory DCs in the lung, highly pathogenic influenza viruses (H7N7, H5N1, and H1N1v) can affect the homing process of DCs into the thymus. In particular, infected respiratory DCs migrated into the thymus, promoting the thymic viral spread and leading to alterations of the antigen presentation, which is essential for the functional development of thymocytes. Thus, infected dendritic cells can interfere with the T-lymphocyte selection processes into the thymus, leading to thymic atrophy [113,114].

It has been also shown in mice that Influenza A virus (IAV) infection led to severe thymic atrophy caused by increased thymic T-cell apoptosis and suppressed proliferation, through a mechanism involving IFN-γ production by NK cells [115].

A Severe Combined ImmunoDeficiency, SCID-humanized mouse model infected with different strains of the Measles virus showed a productive infection occurring in thymic epithelial and myelo-monocytic cells, leading to a rapid depletion of CD4^+^CD8^+^ double positive (DP) thymocytes by apoptosis, and contributing to a long-term alteration of immune responses [116]. Consistent with these observations, in vitro infection of thymic epithelial cells with Measles virus induced terminal differentiation and apoptosis associated with the production of type-1 interferon (IFN-1) [117,118]. In ex vivo human thymus-derived organotypic cultures, thymocytes were susceptible to Measles virus infection with a higher grade of replication in immature CD4^+/^CD8^+^ double positive cells. Furthermore, the susceptibility to infection correlated with both the level of expression of the Measles virus receptor CD150 and the age of thymus donors [119].

Barros et al. showed that TECs express the receptor for HTLV-1 virus and can be infected by this virus, impairing the expression of anti-apoptotic genes, chemokines, and adhesion molecules, even though the expression profile of antigen presentation molecules remained unchanged. Interestingly, infected TEC cells transmitted the HTLV-1 virus to CD4^+^ cells, functioning as a reservoir of the infection [120]. In a SCID-hu mouse, CMV has been found to be able to infect the thymus in vivo [121]. In particular, the majority of virus-infected cells were epithelial cells, mainly localized in the thymic medulla, rather than the cortical region [121]. Type-B Coxsackievirus (CV-B) has been shown to infect TECs in vitro, and to trigger strong production of IL-6, GM-CSF, and leukocyte migration inhibition factor (LIF) in infected cells [122]. In addition, evidence that Coxsackievirus infection of a murine mTEC line induced a dramatic decrease in both *Igf2* gene transcription and IGF-2 production [123] strongly supports the hypothesis that CV-B infection of the thymus could disrupt central self-tolerance to the insulin/insulin-like growth factor family members, contributing to the development of auto-immune diabetes [124]. 

Furthermore, a significant reduction of T-cell Receptor Excision circles, TREC counts, an episomal DNA generated during the re-arrangement of thymic T-cell receptors, and as such a reliable marker for thymus activity, was observed in children hospitalized for respiratory syncytial virus (RSV) infection, as opposed to healthy individuals [125]. This suggests that RSV infection might exert a strong impact on thymus activity, despite the fact that a direct RSV infection of thymus has not been experimentally demonstrated, so far.

Myasthenia gravis (MG) is a prototype autoimmune disease where the muscle weakness is largely induced, and consequent to, the production of autoantibodies, which bind to the muscle postsynaptic junction, disrupting the function and proper activity of acetylcholine receptors (AChR) [134]. To date, it is commonly accepted that the primary site of this autoimmune disorder is the thymus. Although the etiopathogenesis of MG is still unclear, affected individuals show thymic hyperplasia, thymoma, or thymic involution. In MG patients with a hyperplastic thymus, the gland appears to be mainly composed of B-lymphocytes that are either organized into ectopic germinal centers (GCs) or distributed throughout the thymic medulla. Despite this strong morphological evidence, the key molecular factors triggering and promoting the development of MG with thymic follicular hyperplasia remain to be discovered. According to the scientific evidence collected so far, the major MG-dependent thymic alterations affect the fitness and activity of natural regulatory T cells. Likewise, fewer regulatory T cells can be observed in the periphery. In addition, MG thymic effector T cells are less responsive to Treg repression, contributing to the observed pro-inflammatory thymic environment [135]. In general terms, viral or bacterial infections leading to chronic inflammation of the thymus may trigger the development of autoimmunity, thereby contributing to the pathogenesis of MG [136,137]. Consistent with this hypothesis, one of the major candidates as an environmental risk factor for MG is Epstein-Barr Virus, EBV infection [137], and recent works suggest that EBV infection contributes to the pathogenesis of MG within the thymus through a sustained stimulation of TLR-7 and TLR-9, thus, de-regulating innate immunity [138,139]. Furthermore, a recent study shows how the Parvovirus B19 infection is strongly related to thymic hyperplasia and thymic hyperplasia-associated MG [140]. 

## 4. Conclusions and Perspectives

To date, the role of the thymus as a primary lymphoid organ responsible for the generation of mature T cells is well known and has been generally accepted. In view of its function, the microenvironmental compartment of the thymus is extremely complex, characterized by a wide variety of cells and matrix molecules that orchestrate thymocyte maturation into CD4^+^ or CD8^+^ single-positive (SP) T cells. Despite the significant advancements achieved in recent decades, many aspects, including the molecular mechanisms, regulating (both in physiological and pathological conditions) the activity of this gland, and thymocyte differentiation, still need to be further investigated. More specifically, novel cell subsets, including novel immature T cells, thymic epithelial cells (TEC), macrophages, dendritic cells, and fibroblasts within the thymus microenvironment could be identified, as well as novel transcriptional factors, microRNAs, and specific cytokines governing T-cell maturation. 

Among the environmental factors, pathogens can severely affect thymocyte development and migration to other lymphoid tissues, causing severe thymic atrophy and aberrant behavior of peripheral T-lymphocytes, inducing the typical “cytokine storm”, characterized by chronically elevated or excessive production of pro-inflammatory cytokines, associated with chronic inflammation, as observed in Myasthenia gravis. More efforts are required to study thymus development and function upon viral, bacterial, fungal, and protozoan infections, which could unveil novel mechanisms of aberrant thymocyte development operating under the biological pressure of specific infective agents. This aspect would be particularly relevant for envisaging novel therapeutic tools and strategies aimed, for instance, at expanding the thymic stromal compartment and improving thymopoiesis in order to counteract the action of human pathogens.

## Figures and Tables

**Table 1:** Thymus parenchymal populations.

Cell Type	Maturation State/Phenotype	References
Dendritic macrophages	CD45^+^, F4/80^+^, Mac-2^+^	[9,10]
Flat-shaped macrophages	F4/80^+^, CD32/16^+^, Mac-2^−^	[9,10]
Small oval macrophages	Mac-2^+^	[9,10]
Type 1 Conventional dendritic cells	CD11c^high^, MHC II^+^, CD45RA^−^, CD8α^+^	[11,12]
Type 2 Conventional dendritic cells	CD11c^high^, MHC II^+^, CD45RA^−^, SIRPα^+^	[11,12]
Plasmacytoid dendritic cells	CD11c^medium^ MHC II^low^, CD45RA^high^, CD45R^high^	[11,12]
cTECs	CD45^–^, EpCAM^+^, Ly51^+^, K8^+^, K5^–^, K14^–^	[13,14,15,16,17,18]
mTECs	CD45^–^, EpCAM^+^, Ly51^–/low^, CD80^high^, K8^–^, K5^+^, K14^+^	[13,14,15,16,17,18]
Sphere-forming TEC	FoxN1-	[19]
Thymic nurse cells	K5^+^, K8^+^, pH91^+^	[20,21,22]

**Table 2:** Thymic cells.

Cell Type	Phenotype	References
Thymic NK cells	CD127 (IL-7 receptor α)	[3,4,5,6,7,8]
iNKT cells	CD1d-restricted Thymic NK cells	[6,7]
TSP type 1—Fetal BM-derived hematopoietic progenitors as thymus colonizing cells	CD34^high^, CD45RA^high^, CD7^+^	[29]
TSP type 2—Have the capacity to develop into B cells, NK cells and T cells using in vitro co-culture systems	Lin^−^, CD34^+^, CD10^+^, CD24^−^	[30]
TSP type 3—Possess full lymphoid and monocytic potential, but lack erythroid potential	Lin^−^, CD34^+^, CD10^−^, CD45RA^+^, CD62L^high^	[31]
Double Negative 1	CD34^−^, CD38^−^, CD1a^+^	[31]
Double Negative 2	CD34^−^, CD38^+^, CD1a^+^	[31]
Double Negative 3	CD34^+^, CD38^+^, CD1a^+^	[31]
Double Negative 4—Immature single positive	CD3^−^, CD4^+^	[31]
Double Positive	CD3^+^, CD4^+^, CD8^+^	[16,17,19,32,33,34,35]
Single Positive	CD3^+^, CD4^+^, CD8^−^/CD3^+^, CD4^−^, CD8^+^	[31,32,33,34,35,36,37,38]
B-cells	CD19^+^	[23,24,25,26,27,28]

**Table 3:** Surface antigen markers of T-cell development in humans and mice.

	Progenitors	DN1	DN2	DN3	DN4	DP	SP
Human	CD34^high^, CD45RA^high^, CD7^+^	CD34^−^, CD38^−^, CD1a^+^	CD34^−^, CD38^+^, CD1a^+^	CD34^+^, CD38^+^, CD1a^+^	CD3^−^, CD4^+^	CD4^+^, CD8^+^	CD4^+^, CD8^−^ or CD4^−^, CD8^+^
Mouse	Lin^−^, IL-7R^+^, Thy-1^−^, Sca-1^low^ c-Kit^low^	CD117/c-KIT^+^, CD44^+^, CD25^−^	CD117/c-KIT^+^, CD44^+^, CD25^+^	CD117/c-KIT^−^, CD44^−^, CD25^+^	CD117/c-Kit^−^, CD44^−^, CD25^−^	CD4^+^, CD8^+^	CD4^+^, CD8^−^ or CD4^−^, CD8^+^

**Table:** (**a**)

Transcriptional Factor	Cell Type	Function	References
GATA-3	Thymic NK cells	Drive NK development from early thymocyte precursors	[3,4,5]
Notch	DN3 cells	Triggers the β-selection process	[57,58,59]
GATA-3, Tcf7, Bcl11b	T-cells	Drives the early stage of T-cells development	[60,61,62,63,64,65,81,82]
Bcl11b	DN2 cells	Completes the exclusion process, limits non-T-cell fates, inhibits NK lineages, decrease Kit expression	[66,67,68,69,70]
PU.1, Pax5	Immature lymphoid cells	If not inhibited by Gata-3, drive B-cell and myeloid differentiation	[63,64]
Myb, Tcf1	Immature lymphoid cells	Gata-3 positive regulators	[65]
E2A, HEB, Gata-3, Tox, Tcf1, Lef	CD4 cells	Drive CD4 lineage	[71]
ThPOK	CD4^+^/CD8^+^	Drives CD4+ Th fate and prevents the differentiation of thymocytes in CD8+ CTLs	[71]
	Mature T-cells	Blocking the cytotoxic fate of MHC class II-restricted CD4^+^ T cells	[74]
Runx3	CD4^+^/CD8^+^	Abolishes CD4 expression inducing CTL-lineage differentiation	[71]
Runx1, Runx3, Mazr	CD8 cells	Inhibit the expression of ThPOK	[75,76,77,78]
NF-κB	Positive selection survived T-cells	Required for late maturation	[79,80]

**Table:** (**b**)

Transcriptional Factor	Cell Type	Function	References
FoxN1	TEPC	Development of TEPC in mTECs and cTECs; maintains postnatal TEC homeostasis; its lack arrests TEPC differentiation	[82,83,84,85,86]
p63	TEPC	Required in epithelial development of thymus and epidermis; required for proliferation and differentiation	[87,88]
AIRE	CD80^hi^ MHC-II^hi^ mTECs	Critical for thymus development, required for clonal deletion of self-reactive T cells; involved in expression of TRA	[93,94]
Fezf2	mTECs	Regulates TRAs expression different from those of AIRE	[95,96]
Pou2f3	Thymic tuft cells	Essential for the maturation of thymic tuft cells	[54]

**Table 5:** Impact of viruses on thymus.

Virus	Target	Effect	Reference
MLV/HBV/LCMV	TEC/TEC stem cells	Impaired tolerance	[99,100,101,102,103]
HIV/SIV	DP cells, thymocytes	Thymic atrophy; DN to DP block	[104,105,106,107,108,109,110,111,112]
H7N7/HSN1/H1N1	Respiratory DCs in lung before thymic migration	Thymic atrophy	[113,114]
IAV	Thymocytes	Increased INFγ production by NKs; increased T-cells apoptosis	[115]
Measles	TEC/Myelomonocytic cells	Reduced DP cells	[116,117,118,119]
HTLV-1	TEC	Virus transmission to CD4; impaired gene expression	[120]
CMV	mTEC	Infects epithelial cells rather than hematopoietic cells	[121]
CV-B	TEC/mTEC	Increased IL-6/GM-CSF/LIF production; increased IgF2 production and autoimmune diabetes	[122,123,124]
RSV	Thymocytes	Reduced TREC	[125]
